# Evaluation of Risk Scores as Predictive Tools for Stroke in Patients with Retinal Artery Occlusion: A Danish Nationwide Cohort Study

**DOI:** 10.1055/s-0042-1758713

**Published:** 2022-11-30

**Authors:** Marie Ørskov, Henrik Vorum, Torben Bjerregaard Larsen, Flemming Skjøth

**Affiliations:** 1 Department of Cardiology, Aalborg University Hospital, Aalborg, Denmark; 2 Department of Clinical Medicine, Aalborg Thrombosis Research Unit, Faculty of Health, Aalborg University, Aalborg, Denmark; 3 Department of Ophthalmology, Aalborg University Hospital, Aalborg, Denmark; 4 Unit for Clinical Biostatistics, Aalborg University Hospital, Aalborg, Denmark

**Keywords:** Retinal artery occlusion, Prediction, Risk scores, CHA _2_ DS _2_ -VASc score, ESSEN Stroke Risk score

## Abstract

**Purpose** We investigated the 1-year risk of stroke in patients with retinal artery occlusion and evaluated the predictive and discriminating abilities of contemporary risk stratification models for embolic stroke.

**Methods** This register-based cohort study included 7,906 patients with retinal artery occlusion from Danish nationwide patient registries between 1995 and 2018. The study population was stratified according to the number of points obtained in the stroke risk scores: the CHA _2_ DS _2_ -VASc score and the ESSEN Stroke Risk score. The 1-year risk of stroke within strata was evaluated and compared using the cox proportional hazards model. Furthermore, the discrimination of the risk scores as predictive tools for stroke risk assessment was investigated using C-statistics, Brier score, and the index of prediction accuracy.

**Results** The stroke event rate in patients with retinal artery occlusion increased as the score increased for both risk scores, ranging from 3.62 (95% confidence interval [CI]: 2.46–5.31) per 100 person-years to 13.25 (95% CI: 11.78––14.89) per 100-person-years for increasing levels of the CHA _2_ DS _2_ -VASc score and from 3.97 (95% CI: 2.97–5.32) per 100 person-years to 16.43 (95% CI: 14.01–19.27) per 100 person-years for increasing levels of the ESSEN Stroke Risk score. Using a risk score of 0 as a reference, the difference was statistically significant for retinal artery occlusion patients with a CHA _2_ DS _2_ -VASc score of 2 or above and for all levels of the ESSEN Stroke Risk score. The C-statistics for the risk scores was 61% (95% CI: 58%–63%) and 62% (95% CI: 59–64%) for the CHA _2_ DS _2_ -VASc score and ESSEN Stroke Risk score, respectively.

**Conclusion** The results suggested that the use of the CHA _2_ DS _2_ -VASc score and the ESSEN Stroke Risk score was applicable for risk stratification of stroke in patients with retinal artery occlusion, but discrimination was poor due to low specificity.

## Introduction

Retinal artery occlusion is an ocular emergency, often resulting in irreversible vision loss. 1 Retinal artery occlusion is strongly associated with multiple cardiovascular diseases, including stroke. 2 3 4 5 6 7 Stroke is the second leading cause of mortality worldwide, only exceeded by heart disease. 8

The association between retinal artery occlusion and stroke has been investigated in previous studies, in which the risk of stroke in retinal artery occlusion patients has been determined to range between two and nine-fold higher compared with randomly selected controls, depending on time after event, with the highest incidence up to 1 month after the retinal artery occlusion event. 9

Retinal artery occlusion and stroke have been described as equivalent. 10 11 12 13 The retina is a component of the nervous system, during embryonic development the retina arises from the neural tube and is an extension of the diencephalon. 1 14 15 Furthermore, the blood–brain barrier shares common structural and functional features with the blood–retina barrier. 16 17 The retina and the brain share blood supply. The central retinal artery is the first branch of the internal carotid artery, which also supplies the brain with blood. 1 18 In addition, the pathogenesis of retinal artery occlusion and stroke is in both cases thromboembolic. 1 5 19 20

All these shared features support a strong association between retinal artery occlusion and stroke. The prevalence of retinal artery occlusion will increase with the globally ageing population, 21 making optimized management and clear guidelines essential to reduce the disease burden associated with retinal artery occlusion, especially for an important disease such as stroke. Guidelines recommend that patients with retinal artery occlusion are immediately referred for stroke evaluation to reduce the risk of stroke, the evaluation should include brain imaging such as ultrasound (UL), computed tomography (CT), or magnetic resonance (MR) scans. 3 10 11 22 23 24 25 26 27 28 However, among ophthalmologists, not all retinal artery occlusion patients are referred to immediate stroke risk evaluation 25 26 and no specific guidelines specify which patients should be referred.

This study will investigate whether existing risk assessment models can be utilized in patients with retinal artery occlusion to evaluate the 1-year risk of stroke and the need for referral for stroke evaluation.

## Methods

### Data Sources

For this cohort study, data were obtained using different Danish nationwide registries. All residents in Denmark are provided a unique personal identification number at birth or immigration. Using this identification number, data from the included registers can be cross-linked. Three registers were used in this study: first, The Danish Civil Registration System, where information about the date of birth, sex, vital status, and migration can be obtained 29 ; second, The Danish National Patient Register, which includes all hospital admissions along with diagnoses, procedures, and outpatient services, 30 since 1994, the Danish National Patient Register has classified data using the International Classification of Diseases version 10 (ICD-10); and finally, The Danish National Prescription Registry, holding information on individual-level data about all prescription drugs sold in Danish pharmacies. The Danish National Prescription Registry uses the global Anatomical Therapeutic Chemical classification codes. 31

### Study Population and Risk Stratification

The study population included all subjects with a discharge code of retinal artery occlusion (ICD-10: H340, H341, and H342, including subcodes) between January 1, 1995 and December 31, 2018. The date of the diagnosis was defined as the index date. Subjects were excluded from the population if they were <18 years, died on the index date, immigrated to Denmark within the last year, or had inconsistent demographic information.

Baseline characteristics were obtained from the Danish National Patient Register and the Danish National Prescription Registry. Baseline characteristics concerning medication were based on claimed prescriptions within 1 year prior to the index date. Furthermore, the prevalence of scanning was retrieved from the Danish National Patient Registry using procedure codes for CT, MR, and UL scanning of the head or neck.

Risk stratification was performed based on the CHA _2_ DS _2_ -VASc score and the ESSEN Stroke Risk score used on the retinal artery occlusion population evaluated at the time of diagnosis. The CHA _2_ DS _2_ -VASc score was developed for risk stratification of stroke in patients with atrial fibrillation. 32 The CHA _2_ DS _2_ -VASc score ranges from 0 to 9 and is calculated based on points accumulated from prevalent congestive heart failure (1 point), hypertension (1 point), diabetes mellitus (1 point), prior ischemic stroke/systemic embolism/transient ischemic attack (2 points), vascular disease (1 point), age 65 to 74 years (1 point), age ≥75 years (2 points), and female sex (1 point). 33 The ESSEN Stroke Risk score was developed for risk stratification of recurrence in patients with stroke. The ESSEN Stroke Risk score ranges from 0 to 9 and is calculated based on points accumulated from prevalent hypertension (1 point), diabetes mellitus (1 point), myocardial infarction (1 point), peripheral arterial disease (1 point), ischemic stroke or transient ischemic attack (1 point), other cardiovascular events (1 point), age 65 to 75 years (1 point), age >75 years (2 points), and current smoking (1 point). 34 Patients were divided into groups based on the sum of the specified points. Scores of ≥4 points were collapsed due to the small sample size. See Supplementary Table S1 for details on score definitions.

### Outcomes

In this study, the outcome of interest was stroke (ICD-10: I63 and I64). Patients were followed until stroke, the competing event of death or censoring due to emigration, or administrative end of follow-up at December 31, 2018. The follow-up time was 1 year.

### Statistics

Survival analyses were used to assess the risk of stroke in patients with retinal artery occlusion. Baseline characteristics were summarized using descriptive analysis with proportions for categorical covariates and means for continuous variables. In addition, the proportion of retinal artery occlusion patients using platelet aggregation inhibitors or getting a CT, MR, or UL scanning within 48 hours was identified. The Aalen–Johansen estimator was used to estimate the cumulative incidence of medical use 3 months and 1 year after the index date. Additionally, an Aalen–Johansen estimator was used to estimate the cumulative incidence of stroke according to score strata for both risk scores considering death as a competing event.

The crude event rate for stroke was calculated as events per 100 person-years. Subsequently, a Cox proportional hazard ratio model and a Fine and Gray model ( Supplementary material ) were used to calculate the hazard rate ratio and subdistributional hazard rate ratio of stroke according to the risk score level, 35 36 the latter with death as competing event. Crude models were used since the performance of the risk score was investigated. A likelihood ratio test was performed to assess the overall effect of the risk scores compared with the null model.

C-statistics or the area under the receiver operating characteristics-curve provided a measure of the discrimination of the investigated model. 37 To investigate the accuracy of the risk prediction in terms of both discrimination and calibration, the Brier score was calculated. The Brier score is applicable for survival data and is the mean squared difference between the observed status and the predicted risk. A Brier score of 0 would indicate a perfectly calibrated prediction model. 38 39 Afterward, the index of prediction accuracy was determined, which is 1 minus the ratio between the null model and Brier score of the investigated model. 39 40

## Results

A total of 7,906 retinal artery occlusion patients were identified with a mean age of 69.2 years and of which 46.9% were female. Of the included patients, 73.3% had a CHA _2_ DS _2_ -VASc score of 2 or above and 54.8% had an ESSEN Stroke Risk score of 2 or above, suggesting experiencing cardiovascular events prior to their retinal artery occlusion event ( Table 1 ). At baseline 40.7% of the study population had a recent prescription of platelet aggregation inhibitors and after 1 year this percentage reached 65.9% (95% confidence interval [CI]: 64.8–66.9%). Within the first 48 hours of their diagnosis, only 5.9% of the RAO patients had a hospital record of a procedure for scanning of the carotid artery to check for stenosis ( Table 2 ).

**Table 1:** Baseline characteristics of the population comprising patients with retinal artery occlusion

Characteristics	Population
*n*	7,906
Female % ( *n* )*	46.9 (3711)
Age mean (SD)*	69.2 (13.4)
Arterial hypertension % ( *n* )	40.4 (3196)
Diabetes % ( *n* )	14.6 (1157)
Ischemic heart disease % ( *n* )	18.3 (1449)
Peripheral artery disease % ( *n* )	10.8 (856)
Stroke % ( *n* )	11.8 (933)
Atrial fibrillation % ( *n* )	9.0 (713)
Heart disease % ( *n* )	12.8 (1009)
Renal disease % ( *n* )	4.0 (313)
Cataract % ( *n* )	22.8 (1806)
Glaucoma % ( *n* )	5.9 (467)
CHA _2_ DS _2_ -VASc % ( *n* )	
0	9.6 (758)
1	17.0 (1343)
2	20.2 (1600)
3	21.3 (1687)
≥4	31.8 (2518)
ESSEN % ( *n* )	
0	15.1 (1192)
1	30.2 (2386)
2	24.2 (1912)
3	16.1 (1271)
≥4	14.5 (1145)

Abbreviations: SD, standard deviation.

**Table 2:** Treatment characteristics after the index date for retinal artery occlusion diagnosis

Characteristics	% (95% CI)
CT, MR, or UL scan of head or neck within 48 h	5.9 (exact)
Platelet aggregation inhibitors	
Baseline	40.7 (exact)
3 mo after index date	55.6 (54.4–56.7)
1 y after index date	65.9 (64.8–66.9)

Abbreviations: CI, confidence interval; CT, computed tomography; MR, magnetic resonance; UL, ultrasound.

During the 1-year follow-up, a total of 565 stroke events were observed. The overall rate per 100 person-years was 7.99 (95% CI: 7.36–8.68). This incidence rate increased correspondingly with the level of both scores ( *p* < 0.00). An incidence rate of 3.62 (95% CI: 2.46–5.31) per 100 person-years was determined for patients with a CHA _2_ DS _2_ -VASc score of 0, reaching 13.25 (95% CI: 11.78–14.89) per 100 person-years for patients with a score of 4 or above. For the ESSEN Stroke Risk score, the incidence rate was 3.97 (95% CI: 2.97–5.32) per 100 person-years in patients with a score of 0 up to 16.43 (95% CI: 14.01–19.27) per 100 person-years in patients with a score of 4 or above. All incidence rates depending on both the CHA _2_ DS _2_ -VASc score and the ESSEN Stroke Risk score are summarized in Table 3 .

**Table 3:** Stroke in patients with retinal artery occlusion

	Events *n*	Person-time 100 PY	Rate /100 PY (95% CI)	Risk % (95% CI)	Effect HR (95% CI)	*p* -Value
CHA _2_ DS _2_ -VASc						
0	26	7.19	3.62 (2.46–5.31)	3.4 (2.3–4.9)	Ref	
1	71	12.54	5.66 (4.49–7.15)	5.3 (4.2–6.6)	1.56 (0.99–2.44)	
2	92	14.63	6.29 (5.13–7.72)	5.8 (4.7–7.0)	1.71 (1.11–2.65)	
3	97	15.27	6.35 (5.21–7.75)	5.8 (4.7–7.0)	1.72 (1.12–2.65)	
≥4	279	21.06	13.25 (11.78–14.89)	11.2 (10.0–12.5)	3.48 (2.33–5.20)	0.000
ESSEN						
0	45	11.32	3.97 (2.97–5.32)	3.8 (2.8–5.0)	Ref	
1	130	22.08	5.89 (4.96–6.99)	5.5 (4.6–6.5)	1.47 (1.04–2.06)	
2	127	17.09	7.43 (6.24–8.84)	6.7 (5.6–7.9)	1.82 (1.30–2.56)	
3	112	11.00	10.18 (8.46–12.25)	8.9 (7.4–10.6)	2.47 (1.74–3.48)	
≥4	151	9.19	16.43 (14.01–19.27)	13.4 (11.5–15.4)	3.85 (2.76–5.38)	0.000

Abbreviations: CI, confidence interval; HR, hazard rate ratio; PY, person years.

The 1-year cumulative incidence for the groups with no points in the CHA _2_ DS _2_ -VASc score and the ESSEN Stroke Risk score were, 3.4% (95% CI: 2.3–4.9%) and 3.8% (95% CI: 2.8–5.0%), respectively. The cumulative incidence ranged from 5.3 to 5.8% for CHA _2_ DS _2_ -VASc scores of 1 to 3 and reached 11.2% (95% CI: 10.0–12.5%) for a CHA _2_ DS _2_ -VASc score of 4 or above. For the ESSEN Stroke Risk score, the cumulative incidence ranged from 5.5%-8.9% for scores of 1-3 and reached 13.4% (95% CI: 11.5–15.4%) for a score of 4 or above ( Table 3 ). The cumulative incidence for CHA _2_ DS _2_ -VASc scores of 1, 2, and 3 was similar, where the cumulative incidence for the ESSEN Stroke Risk score increased for each stratum.

For the CHA _2_ DS _2_ -VASc score, the hazard rate ratio for score 1 was 1.56 (95% CI: 0.99–2.44) and 3.48 (95% CI: 2.33–5.20) for the score 4 or above, respectively, with score 0 used as reference. The hazard rate ratio for the ESSEN Stroke Risk score range between 1.47 (95% CI: 1.04–2.06) for score 1 and 3.85 (95% CI: 2.76–5.38) for score 4 or above. In a Fine and Gray model conducted to evaluate the score on the risk scale, the effect estimates were comparable between the two models. Estimates of the Fine and Gray model are summarized in Supplementary Table S2 .

The predictive abilities of the two risk scores were investigated using C-statistics, Brier score, and index prediction accuracy score. These were all comparable for both scores. The C-statistics for the CHA _2_ DS _2_ -VASc score and the ESSEN Stroke Risk score, respectively, were 61% (LB-UB: 58–63%) and 62% (LB-UB: 59–64%). The Brier score for the CHA _2_ DS _2_ -VASc score was 2.715 (LB-UB: 2.375–3.051), which yielded an index of prediction accuracy of 0.00. Comparable results were identified for the ESSEN Stroke Risk score with a Brier score of 2.713 (LB-UB: 2.372–3.044) and an index of prediction accuracy of 0.00 ( Table 4 ).

**Table 4:** Predictive performance of stroke in patients with retinal artery occlusion using the CHA _2_ DS _2_ -VASc score and the ESSEN Stroke Risk score

	C-statistics % (LB–UB)	Brier score % (LB–UB)	Index of prediction accuracy score
Null model		2.716 (2.375–3.052)	
CHA2DS2-VASc	61 (58–63)	2.715 (2.375–3.051)	0.0002 (−0.0009–0.0021)
ESSEN	62 (59–64)	2.713 (2.372–3.044)	0.0007 (−0.0004–0.0030)

Abbreviations: LB, lower bound; UB, upper bound.

## Discussion

In this nationwide cohort study of patients with retinal artery occlusion, the risk of a subsequent stroke increased significantly with increasing points in both the CHA _2_ DS _2_ -VASc score and the ESSEN Stroke Risk score. However, the discrimination of the risk assessment models was poor due to the relatively low prevalence of stroke in the population.

The need for specific and effective guidelines in relation to the management of retinal artery occlusion patients is especially important regarding stroke. Worldwide stroke has been ranked the second most common cause of death and the third most common cause of disability. A study of the global disease burden of stroke estimated that the global absolute number of first-time stroke event in 2010 was 16.9 million, with a total of 5.9 million stroke-related death the same year. 41 The burden of stroke-related deaths has similarly been identified in patients with retinal artery occlusion. The mortality rate was investigated in a population of 8,580 participants of older white persons and determined to be higher in patients with retinal artery occlusion compared with controls, with an all-cause mortality of 56% in retinal artery occlusion compared to 30% in controls. 42 The hazard rate ratio of stroke-related mortality was 2.0 (95% CI: 1.1–3.8), which was statistically significant compared to controls. 42 A population of 4,926 participants aged 43–84 years was investigated in both 1999 and 2003. In 1999, it was determined that patients with retinal artery occlusion are three times more likely to experience a fatal stroke compared to controls during an 8-year follow-up period. 43 In 2003, the same research group described the risk of a mention of stroke on the death certificate of patients with retinal artery occlusion to be 2.4 times higher compared with controls without a retinal artery occlusion event during an 11-year follow-up period. 44

**Fig. 1:**
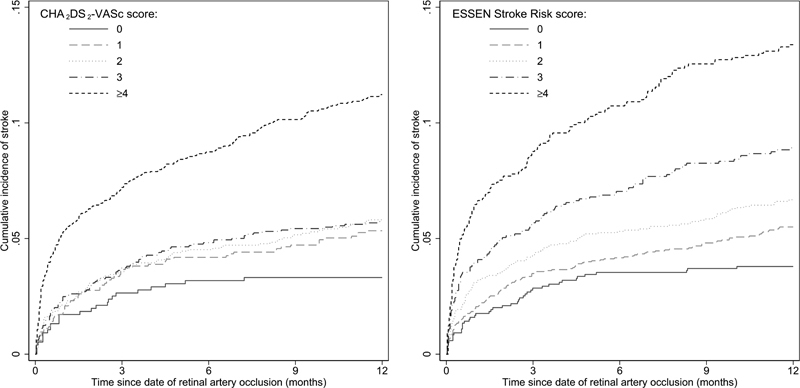
Cumulative incidence of stroke during the 1–year follow-up.

Based on these elevated risks, the need to identify patients with retinal artery occlusion at increased risk of stroke is highly relevant. It is important to emphasize that this study focuses on the stroke risk and whether the included risk scores are able to select patients with retinal artery occlusion who would benefit from early instigation of treatment to prevent subsequent stroke. We found that only 5.9% of the included patients were scanned within 48 hours after their retinal artery occlusion diagnosis ( Table 2 ). Even though it is recommended. For all risk strata, irrespective of score, the risk of stroke is high even within the first month after the occlusion ( Fig. 1 ). This could indicate that a subgroup of patients is at acute risk of stroke and would benefit from an immediate referral for stroke evaluation and prophylaxis treatment. The physician may need a predictive tool to identify the patients at high risk of stroke to refer them for immediate stroke evaluation. Thereby, giving the physician a tool to ensure acute referral for stroke assessment for the more critical patients following their retinal artery occlusion event and when adequate a subacute referral for the less critical patients.

In this study, the CHA _2_ DS _2_ -VASc score was chosen as one of the risk stratification options. Originally, the score was developed as a stroke risk stratification schema for patients with atrial fibrillation. 32 The related CHADS _2_ score and the CHA _2_ DS _2_ -VASc score show comparable clinical utility when predicting stroke. However, the CHADS _2_ score categorizes the largest proportions of patients into the intermediate-risk strata. The CHA _2_ DS _2_ -VASc score on the contrary distributes patients more equally in the different groups, which enables a more specific evaluation of the individual patient. In addition, the CHA _2_ DS _2_ -VASc score identified extremely low-risk patients more precisely compared to the CHADS _2_ score. 32 45

The second risk stratification option used was the ESSEN Stroke Risk score. The ESSEN Stroke Risk score was developed in specific populations constituted of stroke patients in randomized controlled trials and mainly to predict recurrent stroke. 34 Therefore, it would be beneficial to investigate the usages of this risk score in different populations. Retinal artery occlusion and stroke have been described as equivalent diseases, 11 making a retinal artery occlusion population an obvious choice to investigate the predictive properties of the ESSEN Stroke Risk score.

The two risk scores are conceptually similar, with the addition of points corresponding to the presence of risk factors. Furthermore, both risk scores are recognized as useful for the prediction of stroke risk in the predefined populations, which suggests that a comparison of the use of the two risk scores in the retinal artery occlusion population would be relevant. The tendency observed in the Cox model suggested that these risk scores could hold the potential to predict stroke in patients with retinal artery occlusion, supported by the statistically significant likelihood ratio test.

The C-statistics and the individual Brier score for both the risk scores showed acceptable values for prediction models. The C-statistics for the risk scores included in this study were similar to comparable prediction models with C-statistics ranging between 57 and 75%. 32 46 47 However, the index of prediction accuracy showed the prediction performance of the risk scores were close to zero when compared to the null model representing the population prevalence of stroke. The C-statistics and the Brier score estimates the accuracy of a prediction model, whereas the index of prediction accuracy yields a measure considering the discrimination and calibration adjusted for the null model. In this study, the Brier score of the null model was undistinguishable from the Brier scores of the risk stratification models, suggesting that the discrimination of the subgroup of patients experiencing stroke into risk strata with risks between 3% and 14% was quantitatively not enough to yield improvement of the Brier score. Combined, the results suggest that the risk scores stratify patients with clinically relevant increasing risk of stroke; however, even in the strata with the highest risk, the proportion of patients who do not develop stroke is high.

The Brier score and the index of prediction accuracy seems contradictory to the rate, risk, and effect estimated for stroke in patients with retinal artery occlusion, which is due to the different focus for the analyses. The sensitivity of prediction models for stroke are essential since stroke is a serious disease, where it is desirable to exclude false negatives as much as possible. The Brier score does not consider sensitivity or specificity of a model. 48 Therefore, it is important to consider the clinical aspect when analyzing the data.

The CHA _2_ DS _2_ -VASc score was developed to risk stratify for stroke in patients with atrial fibrillation. However, atrial fibrillation mainly causes cardiogenic embolic strokes. 49 50 Retinal artery occlusion is associated with atherothrombotic embolization originating from the carotid arteries, and cardiogenic embolization is not a primary risk factor. 5 51 52 This suggests a difference between the two diseases and may explain why the CHA _2_ DS _2_ -VASc score did not show the same results in patients with retinal artery occlusion as in patients with atrial fibrillation. To accommodate the need for stroke risk stratification in patients with retinal artery occlusion, it would be valuable to identify a prediction model with accurate probabilistic abilities. The probabilistic abilities may be improved if the risk score was constituted more specifically of atherosclerotic factors, since retinal artery occlusion is predominantly embolic. 53 Furthermore, ophthalmologic factors could be considered as well, since retinal artery occlusion have been associated with other ophthalmologic diseases. 52

## Limitations

This study is limited by its observational design and the use of ICD coding. However, the components of the CHA _2_ DS _2_ -VASc score have been validated in the Danish registries with positive prediction values of >90%. 54 Errors in the ICD coding result in random variation, which especially is problematic in smaller populations. To reduce the possibility of random variation influencing the analyses performed during this study, the highest CHA _2_ DS _2_ -VASc score group was collapsed to ensure larger groups.

Another limitation is the inclusion of a patients using antithrombotic treatment since treatment will reduce the risk of stroke. This could result in an interpopulation variation, including individuals with different risks for stroke, making the population incomparable. However, due to the large proportion of individuals using antithrombotic treatment, it was assessed that the interpopulation variation would not influence the results significantly. No guidelines are available for the management of patients with retinal artery occlusion; however, based on the included data, it was evident that the majority of these patients use antithrombotic treatment, which may have an impact on the risk score level compared to an untreated population. Furthermore, patients with retinal artery occlusion may be managed differently in other countries, which could influence the transferability of this study.

The population assessed at the index date can acquire comorbidities included in the score over time, which would change their score and hence their risk of stroke. 55 This dynamic nature of stroke risk is not considered when conducting analyses during this study.

## Conclusion

The risk of stroke was associated with both increased CHA _2_ DS _2_ -VASc score and ESSEN Stroke Risk score. However, the association was stronger between an increasing ESSEN Stroke Risk score and stroke in patients with retinal artery occlusion compared to an increasing CHA _2_ DS _2_ -VASc score. The predictive probabilities of both the risk scores did not support the use of either score for stroke risk stratification in retinal artery occlusion patients.

## Notes

**What is Known on this Topic?**

- Retinal artery occlusion and stroke have been described as equivalents.
- Referral to a stroke clinic of patients with retinal artery occlusion to undergo immediate evaluation of stroke risk is recommended. However, ophthalmologists do not routinely refer patients with retinal artery occlusion to specialized neurological investigation.
- There are no available guidelines on which patients with retinal artery occlusion that should be referred.

**What Does this Paper add?**

- Increasing levels of the CHA _2_ DS _2_ -VASc score and the ESSEN Stroke Risk score were associated with increasing risk of stroke in patients with retinal artery occlusion. The risk of stroke was high even in strata with no or few risk factors.
- The ESSEN Stroke Risk score was found qualitatively superior for risk stratification compared to the CHA _2_ DS _2_ -VASc score.
