# Supplementary material for: Evaluation of Risk Scores as Predictive Tools for Stroke in Patients with Retinal Artery Occlusion: A Danish Nationwide Cohort Study
Source: TH Open. 2022 Nov 30;6(4):e429–36. doi: 10.1055/s-0042-1758713 (PMC9713298; doi:10.1055/s-0042-1758713)
Supplement: Supplementary file 1 — Supplementary Material [file 10-1055-s-0042-1758713-s22070032.pdf]

Supplemental Material

Supplementary Table S1 Risk score definitions

	CHA2DS2-VASc score	
**Risk factor**	**ICD-10 and ATC codes**	**Score**
Congestive heart failure/LV dysfunction	I110 I130 I132 I420 I50 C03C C09	1
Hypertension	I10 I11 I12 I13 I15 C02A C02B C02C C02DA C02L C03A C03B C03D C03EA C03X C07C C07D C08G C09BA C09DA C09XA52 C02DB C02DD C02DG C04 C05 C07 C07F C08 C09BB C09DB C09	1
Age >75 y		2
Diabetes mellitus	E10 E11 E12 E14 H360 O240 O241 O242 O243 A10	1
Stroke/TIA/thromboembolism	I63 I64 G45 I74	2
Vascular disease	I21 I23 I700 I702 I708 I709 I739	1
Age 65–74 years		1
Sex (female)		1
	**ESSEN Stroke Risk score**	
**Risk factor**	**ICD-10 and ATC codes**	**Score**
Age 65–74 y		1
Age >75 y		2
Diabetes Mellitus	E10 E11 E12 E14 H360 O240 O241 O242 O243 A10	1
Hypertension	I10 I11 I12 I13 I15 C02A C02B C02C C02DA C02L C03A C03B C03D C03EA C03X C07C C07D C08G C09BA C09DA C09XA52 C02DB C02DD C02DG C04 C05 C07 C07F C08 C09BB C09DB C09	1
Myocardial infarction	I21 I23	1
Other cardiovascular event	I20 I22 I24 I25 I30 I31 I32 I33 I34 I35 I36 I37 I38 I39 I40 I41 I42 I43 I44 I45 I46 I47 I49 I50 I51 I52 FNG FNA FNC FND FNE	1
Peripheral cardiovascular events	I70 I71 I72 I73 I74 I77	1
Smoking	C34 J40 J41 J42 J43 J44 J45 J46 J47 J60 J61 J62 J63 J64 J65 J67 J684 J701 J703 J841 J920 J921 J982 J983	1
Stroke/TIA	I63 I64 G45	1

Abbreviations: LV, Left ventricular; TIA, transient ischemic attack.

 © 2022. The Author(s).

Supplementary Table S2 Fine and Gray analysis of stroke risk in retinal artery occlusion patients

	Risk<br>SHR (95% CI)
CHA_2DS_2-VASc	
0	Ref
1	1.55 (0.99-2.43)
2	1.69 (1.10-2.62)
3	1.69 (1.10-2.61)
≥4	3.38 (2.26-5.05)
ESSEN	
0	Ref
1	1.45 (1.04-2.04)
2	1.79 (1.27-2.52)
3	2.41 (1.70-3.41)
≥4	3.71 (2.66-5.18)

Abbreviations: CI, confidence interval; SHR, subdistributional hazard rate ratio.

 © 2022. The Author(s).
